# A commentary on ‘Comparative effectiveness of noninvasive therapeutic interventions for myofascial pain syndrome: a network meta-analysis of randomized controlled trials’

**DOI:** 10.1097/JS9.0000000000001678

**Published:** 2024-05-20

**Authors:** Weijing Fan, Jian Wang

**Affiliations:** Shuguang Hospital Affiliated to Shanghai University of Traditional Chinese Medicine, Shanghai, People’s Republic of China

*Dear Editor,*

We read the recent article ‘Comparative effectiveness of noninvasive therapeutic interventions for myofascial pain syndrome: a network meta-analysis of randomized controlled trials’ by Liu *et al*.^1^ with great interest, which evaluates the comparative effectiveness of various noninvasive therapeutic interventions for myofascial pain syndrome (MPS). The study’s findings suggest that manual therapy, laser therapy, and extracorporeal shock wave therapy are effective in reducing pain intensity and disability associated with MPS are significant for clinical practice. However, we have a few considerations and suggestions that we would like to bring to your attention.

Primarily, Tuina as a key manual therapy component, is well-studied in China^2^. The authors didn’t search Chinese databases, potentially missing valuable data. Future research should include searches in CNKI, Wanfang Medicine, and VIP databases.

Secondly, the authors claimed adherence to PRISMA guidelines, yet the study lacked sensitivity analysis. It’s suggested that reporting standards be further enhanced^3^.

In addition, variations in intervention duration and outcome measurement times across studies can introduce significant heterogeneity. Subgroup analyses and regression analyses based on treatment durations and observation points are advised.

Finally, although the results of this study are positive, the limited study count, neglect of sparse data, and repeated testing in standard meta-analyses risk false positives. Conducting a trial sequential analysis (TSA) is suggested to mitigate these concerns^4^.

## Ethical approval

Not applicable.

## Consent

Not applicable.

## Sources of funding

No fundings were received for this research.

## Author contribution

W.F.: methodology, formal analysis, and writing – original draft; J.W.: conceptualization, methodology, supervision, and writing – review and editing.

## Conflicts of interest disclosure

The author declares no conflict of interest.

## Research registration unique identifying number (UIN)

Not applicable.

## Guarantor

Jian Wang.

## Data availability statement

All data generated or analyzed during this study are included in this article. The data are available from the corresponding author upon reasonable request.
